# Comparative Effects of Isokinetic Training and Virtual Reality Training on Sports Performances in University Football Players with Chronic Low Back Pain-Randomized Controlled Study

**DOI:** 10.1155/2020/2981273

**Published:** 2020-06-16

**Authors:** Gopal Nambi, Walid Kamal Abdelbasset, Shereen H. Elsayed, Saud M. Alrawaili, Ahmed M. Abodonya, Ayman K. Saleh, Tamer E. Elnegamy

**Affiliations:** ^1^Department of Health and Rehabilitation Sciences, College of Applied Medical Sciences, Prince Sattam Bin Abdulaziz University, Al-Kharj, Saudi Arabia; ^2^Department of Physical Therapy, Kasr Al-Aini Hospital, Cairo University, Giza, Egypt; ^3^Department of Rehabilitation Sciences, Faculty of Health and Rehabilitation Sciences, Princess Nourah Bint Abdulrahman University, Riyadh, Saudi Arabia; ^4^Department of Anesthesia and Intensive Care, Faculty of Medicine, Al-Azhar University, Cairo, Egypt; ^5^College of Medicine, Prince Sattam Bin Abdulaziz University, Al-Kharj, Saudi Arabia; ^6^Department of Orthopedics, Faculty of Medicine for Girls, Al-Azhar University, Cairo, Egypt

## Abstract

**Objective:**

The objective of this study is to find and compare the effects of isokinetic training and virtual reality training on sports performances in university football players with chronic low back pain.

**Design:**

This is a randomized, double-blinded controlled study.

**Methods:**

The study was conducted on 45LBP participants at university hospital. First group (*n* = 15) received isokinetic training, second group (*n* = 15) received virtual reality training, and the control group (*n* = 15) received conventional training exercises for four weeks. Clinical (pain intensity and player wellness) and sports performance (40 m sprint, 4 × 5 m sprint, submaximal shuttle running, countermovement jump, and squat jump) scores were measured at baseline, after 4 weeks, 8 weeks, and 6 months.

**Results:**

Four weeks following training VRT group shows more significant changes in pain intensity and player wellness scores than IKT and control groups (*p* ≤ 0.001). Sports performance variables (such as 40 m sprint, 4 × 5 m sprint, submaximal shuttle running, countermovement jump, and squat jump) scores also show significant improvement in VRT group than the other two groups (*p* ≤ 0.001).

**Conclusion:**

Overall, our study suggests that strength training through virtual reality training protocol improves pain and sports performances than isokinetic training and other conventional trainings in university football players with chronic low back pain.

## 1. Introduction

Football has become one of the world's leading team events; according to FIFA's survey, there are 265 million people actively participating in this game around the world. Increase of the number of players also increases the number of sports injuries, which was noted especially in the lower back region (47%) [1]. Low back pain (LBP) is considered to be the major disability affecting this game and the pain is associated with trunk balance control [2]. Recent studies report that an injury to the muscles and proprioceptors in the trunk during the sports activities affects the trunk balance control mechanisms [3]. The decline in trunk balance control may occur due to technological development, abnormal physical activity, pathological changes, and poor training in sports. These factors finally lead to low back pain in the later stages [4, 5]. Participation in football is usually associated with risk of back injuries and grossly affects the activities of daily living and leads to poor quality of life [6]. Hence, various injury prevention and postinjury rehabilitation programs have been formulated to prevent and treat such sports injuries [7–9]. Generally, sports physiotherapists and coaches are providing and adopting such training on and off the field to the players [10].

Ho CW et al. observed that the trunk muscles of subjects with chronic LBP were weaker than normal healthy subjects [11]. It is proved clinically that isokinetic exercise has significant consistent results in mechanical LBP and found the positive correlation between trunk muscle imbalance and low back pain dysfunction [12]. Usually, in clinical studies, the effectiveness of different exercise training protocols and fitness protocols in LBP was evaluated by measuring the core muscle strength [13]. The newly developed isokinetic trunk device is a tool which precisely measures the strength of the core muscles in low back pain subjects. The device was also used as training (isokinetic training (IKT)) and rehabilitating tool for improving the muscle strength in various musculoskeletal conditions [14]. Moreover, operating this device requires a trained person, a suitable place, and a particular appointment time in the sports set-up. Hence, there is lack of studies in the current sports field to analyze its effect on football players with chronic low back pain.

Virtual reality training (VRT) is different type of training commonly used for balance problems in treating musculoskeletal conditions. It uses different virtual games with visual and auditory biofeedback [15, 16]. VRT is used widely due to the fact that the treatment session becomes more interesting which reduces the difficulty of rehabilitation and also increases the safety of the participants. The real scientific physiological advantage of VRT is that this training permits the nervous system for neuroplastic changes and neural organization and transferring into the muscular system for new motor learning [17, 18]. In few studies, there was a significant difference in the clinical outcomes among the subjects who had undergone conventional balance training and VR training in neurological subjects [19, 20]. This way of virtual reality training could also be recognized as an optimum treatment for musculoskeletal conditions too.

Altogether, the knowledge about the effective implementation of IKT and VRT and its effects on the sports performances of football players suffering from chronic low back pain is lacking. Therefore, the aim of the study is to find and compare the effects of isokinetic training and virtual reality training on sports performances in university football players with chronic low back pain. Comprehensive understanding of the relation between sports training and sports performance promotes this clinical condition in a positive way. Hence, these types of sports trainings should be able to modify the risk and reduce the impact of future consequences in football.

## 2. Materials and Methods

### 2.1. Trial Design

The study was a double-blinded randomized control study, and the subjects were randomized and allocated equally according to computer random table method in three groups. Forty-five (*N* = 45) subjects were randomized in the study and allocated to isokinetic training IKT-G (*n* = 15), virtual reality training VRT-G (*n* = 15), and Control-G (*n* = 15) groups. The study was approved by the Departmental Scientific Ethical Committee with reference no. RHPT/019/011 and was conducted according to the ethical guidelines of the declaration of Helsinki 1964 and declaration of Tokyo, 1975. It was executed transparently and presented in accordance with CONSORT guidelines.

The study was executed in the Department of Health and Rehabilitation Sciences, Prince Sattam bin Abdul Aziz University, Al-Kharj, Saudi Arabia. Participants were recruited from the University Hospital and King Khalid Hospital, Al-Kharj, Saudi Arabia. Sports therapist at the department evaluates the participants for participating in the study according to the eligibility criteria.

### 2.2. Patient Involvement

In the initial phase, all the participants were instructed and explained about the research problems, study design, intervention procedures, outcome measures, study duration, harms, and benefits of the research through study information form. Subjects who read and consented to participate in the study were involved in primary screening for selection. We intend to generalize the main results to the study participants and require the subjects and public involvement in the development of an appropriate method of application.

### 2.3. Participants

In order to take part in the study, the subjects have to agree to participate in the study and to sign the informed consent approved by the ethical committee. Inclusion criteria for selection of the subjects were as follows: university male football players in the age group of 18–25 years, chronic (≥3 months) LBP, and 4 to 8 pain intensity in visual analog scale which were included. Participants with severe musculoskeletal, neural, somatic, and psychiatric conditions, waiting for spine surgery, having alcohol or drug abuse, and involving in other weight and balance training programs were excluded from the study. Participants with other soft tissue injuries, fracture at the lower limbs and pelvic bone, and deformities were also excluded from the study.

### 2.4. Interventions

**Figure 1:**
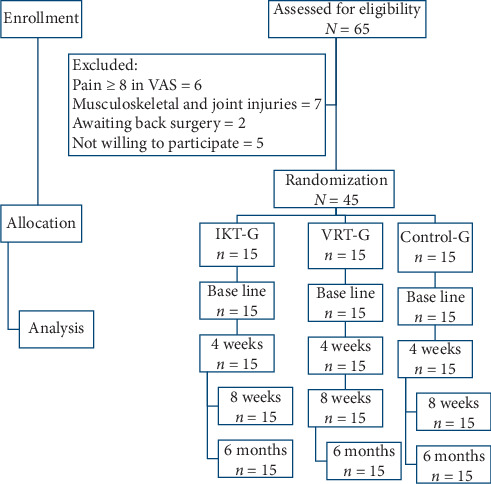
Flow chart showing the study details.

The IKT-G, VRT-G, and Control-G consist of 15 subjects in each group. The 4-week rehabilitation protocols for the three groups were accepted by the ethical committee. The rehabilitation protocol was carried out by an experienced and trained physiotherapist with five years' experience. This protocol specially laid stress on trunk balance training and also they were instructed and advised to exercise at home. We excluded six participants with excruciating pain (≥8 in VAS scale), seven participants with other musculoskeletal and joint injuries, two with awaiting surgery and five who were not willing to participate in the study (Figure 1).

In IKT group before isokinetic training, the subjects were asked to perform five-minute warm-up followed by slow stretching of back extensors and flexors. The subject is asked to be in isokinetic dynamometer (Biodex Corporation, New York, USA) in a vertical standing position. The knees were flexed slightly at 15 degrees, and the fixation straps were tied around the popliteus, thigh, pelvis, chest, and scapula to prevent the tricky movements. Keep the trunk to maintain the range of motion of 10° of extension and 80° of flexion. The axis of the dynamometer was aligned with the intersection point of the midaxillary line and the lumbosacral junction which is exactly 3.5 cm below the crest of iliac bone. The lever arm was customized according to the length of the subject's trunk and the resistance was given anteriorly and posteriorly to the trunk. The required modifications and procedures were done as per the user's manual to reduce the risk. The trunk was tested from −10° of extension to 80° of flexion 0 degree which are considered as neutral.

The subjects were trained for familiarization in the exercise by showing model video clips and allowing them for practice attempts. Once they mastered in the training, they were allowed to perform the exercise at an angular speed of 60 degrees/second, 90 degrees/second, and 120 degrees/second with 15 repetitions of 3 sets. Rest between each set of 30 seconds and between each pace of 60 seconds has been given. The training was given 5 days per week for 4 weeks. The subjects were monitored and instructed throughout their training by a supervisor. The outcome parameters were assessed by different examiner, who was experienced in handling isokinetic devices [21].

VRT-G group received virtual reality balance training with (ProKin system PK 252 N Technobody, pelvic module balance trunk MF, Italy) focusing on the balance of core stability muscles. Training was given to all participants in this group. Personal education about the procedures of executing the game helped the subjects to get the idea about VRT. Training was given in the sitting position which challenges balance activities of the participant. The game which was used in the current study was shooting game, which consists of the subject sitting on the virtual platform and visualizing the game in the display screen. The game is executed and controlled by moving the trunk back and forth and left and right according to the signs. Participant can perform all the six movements of their spine within their pain limits. The level of difficulty of the exercises was increased by graded activity, in which the activities were gradually getting difficult and harder as to participant in more muscle activity and movement.

The level of difficulty in this game is defined by the number of enemies, angle of throw, frequency of shoot, frequency of flashing of enemies, and number of balls appearing around the participant. These five parameters can increase or decrease the difficulty level by increasing or decreasing these parameters. This training has to be continued for 30 mins in each session for 5 days in a week for 4 weeks [22].

The Control-G group focused on conventional balance training for core muscles. The training includes active isotonic and isometric exercise for abdominal muscles (internal oblique, external oblique, transverse abdominis, and rectus abdominis), deep abdominal muscles (psoas major, psoas minor, iliacus, and quadratus lumborum), and back muscles (erector spinae, transverse spinalis, interspinalis, and intertransverse). They perform these exercise 10–15 reps/day for 5 days per week for 4 weeks. Stretching should focus on each muscle group for 3 repetitions for 10 seconds per muscle group (hamstring, hip flexors, and lumbar extensors).

A home-based exercise protocol was prescribed to all the subjects to perform at home. All the subjects in three groups undergone hot pack therapy for 20 mins and ultrasound with a frequency of 1 MHz and intensity of 1.5 W/cm^2^ in continuous form for five minutes [23].

### 2.5. Outcome Variables

Pain intensity: this was measured by visual analog scale (VAS) which consists of 10 cm horizontal line representing one end with “no pain at all” and the other end with “as bad as possible it could be.” Each subject was asked to enter in the line as per his pain perception and the score is measured by the distance on the line. The reliability and validity of VAS in application of musculoskeletal conditions were good [24].

### 2.6. Player Wellness

The wellness of the player was measured by player wellness questionnaire in which the subject fills the 5 items (fatigue, sleep quality, muscle soreness, stress, and mood) in the questionnaire. The subject was asked to score on the 5 point Likert scale where 1 indicates very poor and 5 indicates very well. Therefore, the additions of all 5 items provide the score between 5 and 25 [25].

### 2.7. Sprint Performance

1. 40 m sprint performance: the subjects were instructed to do 10 minutes of warm-up and asked to run for 40 meters with photocell timer (Microgate, Italy) placed at 40 m. Three attempts have been made with 5 minutes of rest period, and the best result is considered for data analysis.
2. 4 × 5 m sprint (S 4 × 5 m): this test was done with five cones which were set at 5 meters apart and the photocell timer (Microgate, Italy) was placed at the beginning and end points. This test required frequent directional changes, where the subject started from the beginning point (cone 1) and ran 5 meters to the point 1 (cone 2), where he made a 90° turn to left and ran for 5 meters to point 2 (cone 3); then, he took a second of 90° turn to the right and ran for 5 meters to point 3 (cone 4). From point 3, he took 180° turn to left and reached the last cone (cone 5) [26].
3. Submaximal shuttle running: the mechanical loading of the subjects was measured by submaximal shuttle running test. It was measured by MEMS device (Colibrys, Tokyo, Japan) and the device was worn in interscapular region. Each subject was asked to do the shuttle run for 20 meters continuously for five minutes with average speed of 12 km per hour. Anterior/posterior, mediolateral, and vertical measurements were recorded [27].

### 2.8. Jump Performance

1. Countermovement jump (CJ): the subjects were instructed to keep the hands placed on the hips and asked to jump to a self-selected depth. They were asked to jump as much as possible without hip or knee flexion during the flight phase.
2. Squat jump (SJ): the subjects were asked to maintain self-selected depth for four-second count and asked to jump as much as possible with hip or knee flexion during the flight phase.

Each jump was performed for 4 times with 30-second rest. All measurements (height, force, and velocity) were measured with optical timing system (Quattro Jump, Switzerland) which is a reliable and valid tool to measure the jump height [28].

### 2.9. Sample Size

Through preliminary pilot study, the subjects required for the study in each group were 12 calculated by assuming 80% power with 20% changes in pain intensity (VAS) with the standard deviation of 2 and significance level of 5%.

### 2.10. Randomization

An individual who is not involved in the data collection was used for randomization. The subjects enter “IKT-G, VRT-G, and Control-G” following simple random table in 1 : 1 : 1 ratio in three groups. All the prospective subjects who fulfilled the eligibility criteria were allowed to participate.

### 2.11. Blinding

Due to the design and settings of the study, it is not possible to blind the treating therapist involved in the study. The subject and the therapist assessing the outcomes at baseline, after 4 weeks, 8 weeks, and 6 months were blinded. Hence, the treating and assessing therapists were different persons and the assessing therapist remains blinded to the subject's treatment group assigned at all times. Subjects were instructed not to disclose the study procedures and treatment protocol with fellow subjects and the assessing therapist.

### 2.12. Statistical Analysis

Subject demographic characteristics were measured to decide the study homogeneity using Levene's test. Outcome data were presented as mean and standard deviation and repeated measures of ANOVA were performed to determine significant difference within the groups. One-way ANOVA test was used for comparison between the groups and the statistical significance level was set at *p* < 0.05. SPSS software (version 20.0) SPSS Inc., Chicago, Illinois, USA, was used for all statistical analyses.

## 3. Results

### 3.1. Participants

Out of 65 participants screened, 45 were selected and allocated equally (*n* = 15) into IKT, VRT, and control group as per the selection criteria. The intention to treat analysis method was presumed in this study but all the participants completed the study with no drop-out. Descriptive demographic analysis of characters such as age, height, weight, and BMI was measured in all three groups at baseline and presented as mean and standard deviation. The one-way ANOVA test shows no significant difference (*p* > 0.05) between these characters in the groups which indicate study homogeneity. Moreover, the clinical parameters such as VO_2_ peak, heart rate, years of playing, and duration of injury were also measured to find the eligibility to participate in the exercise training program. These clinical parameters also show no significant difference (*p* > 0.05) between the groups at baseline (Table 1).

### 3.2. Pain Intensity and Player Wellness

**Figure 2:**
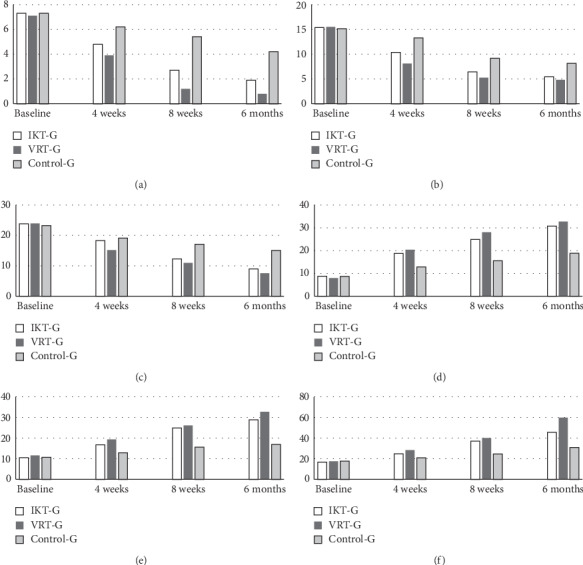
Mean values of pain intensity, 40 m sprint, 4 **×** 5 sprint, shuttle running A/P, M/L, and vertical scores in IKT, VRT, and control group. (a) VAS. (b) 40 m sprint. (c) 4 × 5 sprint. (d) Shuttle running A/P. (e) Shuttle running M/L. (f) Vertical shuttle running.

The baseline scores between IKT, VRT, and control group of pain intensity (VAS) and player wellness have not shown any statistical difference (*p* > 0.05), which represents the homogenous population. Intergroup analysis between IKT, VRT, and control group at 4 weeks, 8 weeks, and 6 months of follow-up shows significance difference (*p* ≤ 0.001) after 4 weeks of training. Moreover, the intragroup analysis of IKT, VRT, and control group shows significance difference (*p* ≤ 0.001) which means that each group has considerable amount of improvement (Table 2). Tukey's post hoc analysis and percentage of improvement between the groups reported that VRT group has more reduction in pain (Figure 2) and improvement in player wellness than IKT and control groups.

### 3.3. 40 m Sprint, 4 × 5 m Sprint, and Submaximum Shuttle Running

The components of sports performance analysis such as 40 m sprint, 4 × 5 m sprint, and submaximum shuttle running were measured before and after 4 weeks of training in all three groups. The follow-up measurements like after 8 weeks and 6 months were also measured to know the short and intermediate effects of these trainings. There was no statistically significant difference (*p* > 0.05) between the three groups at baseline. Four weeks following different training protocols, the participants were measured for the running performance which shows statistically significant difference (*p* ≤ 0.001) between the groups. The eight-week and six-month follow-up measurements also show the difference (*p* ≤ 0.001) between the groups which describe the running effect of IKT and VRT training on sports performance (Table 2). Tukey's post hoc analysis and graphical representation show more tendencies in improvement towards VRT group than IKT group (Figure 2).

### 3.4. Countermovement Jump and Squat Jump

**Figure 3:**
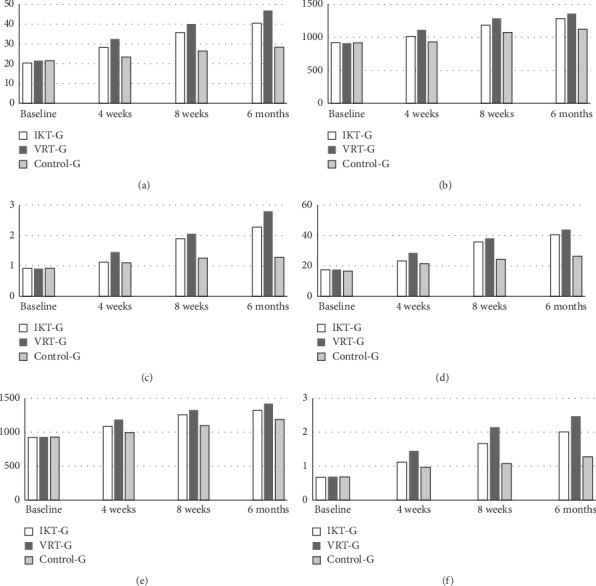
Mean values of counterjump (height, force, and velocity) and squat jump (height, force, and velocity) scores of IKT, VRT, and control group. (a) Counterjump height. (b) Counterjump force. (c) Counterjump velocity. (d) Squat jump height. (e) Squat jump force. (f) Squat jump velocity.

The jump performances such as countermovement jump and squat jump were measured at baseline, after training at 4 weeks, and various intervals like 8-week and 6-month follow-up. There were no statistically significant differences (*p* > 0.05) between the groups in both jump performances at the baseline evaluation. After 4- week training, the analysis between the groups shows significant difference (*p* ≤ 0.001) between the groups in CJ and SJ variables. It is also observed that there is statistically significant difference (*p* ≤ 0.001) between IKT, VRT, and control group in both jumping variables at 8-week and 6-month follow-up measurements (Table 3). However, the percentage of improvement between the groups at 6 months shows more tendencies in improvement towards VRT group than IKT and control groups (Figure 3).

## 4. Discussion

The main objective of this study is to find and compare the different effects of isokinetic training and virtual reality training on sports performances in university football players with chronic LBP. In this study, the pain intensity and player wellness improved significantly in VRT group when compared to IKT and control group. In 40 m sprint, 4 × 5 m sprint, and submaximal shuttle running, VRT group has shown more potential improvement in sports performance and positive changes in the evaluation than the other two groups. Moreover, all the three groups showed substantial improvement in countermovement jump and squat jump after different types of exercise training but little more positive tendencies towards VR training.

### 4.1. Isokinetic Training

In this study, the isokinetic training was given at different angular velocities such as 60 deg/s, 90 deg/s, and 120 deg/s with high peak torque. Calmes et al. observed that training at different angular velocities and high peak torque will improve the trunk muscle strength and flexors/extensors ratio in athletes [29]. These biomechanical changes may clinically reduce the pain and improve the wellness of the football players with chronic low back pain. The current reports on these clinical changes were supported by Ben Moussa Zouita et al. and Zouita et al. and also said that improving trunk muscle strength is the key role in preventing further injuries in the back [30, 31].

We also found little response in 40 m sprint, 4 × 5 m sprint, and submaximal shuttle running after isokinetic training. Training the trunk muscles is an important factor in improving the sports performance in different physical activity and sports. However, it is proved that IKT training in chronic LBP has positive impact on sprint performances and this is in accordance with Hibbs et al. [32]. Moreover, we also looked at the role of isokinetic training on jump performance in football players with chronic LBP. Our study shows some improvement in CJ and SJ after isokinetic training. The reports found considerable improvement in height, force, and velocity after IK training; these changes will be positively helpful in improving sports performance in LBP subjects which is in agreement with the study by Van Damme et al. [33]. The available study on isokinetic training suggests that balanced controlled exercise will further reduce the joint injury and improve the regeneration process which has positive correlation with the sports performance [34].

### 4.2. Virtual Reality Training

The study analyzed the effects of IKT, VRT, and conventional training on pain intensity and player wellness in chronic LBP subjects. This study provides the report that VRT reduces the pain intensity by changes in the inflammatory process than the other two groups. It was noted that greater concentrations of proinflammatory markers such as CRP, TNF-*α*, and IL-6 were present in chronic LBP subjects. VR training induces the players' overall energy expenditure which is higher than the other two groups and results in alterations in inflammatory markers [35]. The possible mechanism behind pain modulation effect of VRT has been suggested to be the release of anti-inflammatory cytokines such as IL-2 and IL-4 receptor antagonist in VRT [36].

Our study also found that VRT group shows significant improvement in sprint and jumping performance than SMT and control groups. VRT increases the activity of the human sensory system and enhances the motor activity, which increases the strength and power of the targeted group of muscles. It uses the real-time feedback information to complete the task and provide the subjects' progress quickly to the next level in the games, which directly fastens the sports performance [17, 37]. Moreover, the games in virtual reality training activate the experience related brain plasticity through the changing environment. Increasing the level of difficulty in the games improves the attention, concentration, memory, and working capacity of these muscles, which were in agreement with [38] but against [39].

The greater strength of this study was sample homogeneity; hence, the reports of this study can be generalized to the sports specific population. This study provided the comprehensive understanding of the relation between different sports training (isokinetic training and virtual reality training) and sports performances in football players suffering from chronic low back pain. The results of this study promoted this clinical condition in a positive way in sports rehabilitation field. Moreover, these types of advanced sports trainings will modify the risks and reduce the impact of future consequences in football.

Also, few limitations have been observed during the implementation of this study. First, this study did not measure and calculate the isokinetic parameters such as concentric, eccentric muscle strength of trunk flexors and extensors. Secondly, the association between the clinical and sports performance characters in chronic LBP after different training protocols has not been analyzed. Finally, follow-up measurements were not taken in a long-term basis, which could have been measured. Therefore, the future studies should involve analyzing the isokinetic parameters in chronic LBP subjects. Also, further researches should try to find the physical and molecular mechanism behind the neuromechanical and anti-inflammatory effect of VRT in chronic LBP subjects.

## 5. Conclusion

Overall, our study suggests that strength training through virtual reality training protocol improves pain and sports performances than isokinetic training and other conventional trainings in university football players with chronic low back pain. Including VRT program in rehabilitation program shows beneficial changes in pain intensity and sports performance in chronic LBP. Still, virtual reality training is relatively presumed as a new training protocol for different sports injuries in different games. Thus, future studies can be done to investigate the different effects of virtual reality training on different sports injuries in different games, which can be questioned and warranted.

## Figures and Tables

**Table 1:** Mean and standard deviation of demographic variables of IKT, VRT, and control group.

No.	Variable	IKT-G	VRT-G	Control-G	*p* value
1	Age (y)	20.23 ± 1.6	21.25 ± 1.2	20.78 ± 1.6	0.253^*∗*^
2	Height (m)	1.68 ± 0.14	1.65 ± 0.18	1.68 ± 0.15	0.865^*∗*^
3	Weight (kg)	65.6 ± 1.4	66.2 ± 1.4	65.5 ± 1.5	0.442^*∗*^
4	BMI (kg/m^2^)	23.2 ± 1.5	22.8 ± 1.6	23.2 ± 1.4	0.754^*∗*^
5	VO_2_ peak (mL/kg/min)	37.3 ± 3.8	38.2 ± 3.5	37.3 ± 3.6	0.784^*∗*^
6	HR (beats/min)	165 ± 5.8	168 ± 5.2	167 ± 5.5	0.407^*∗*^
7	Years of playing (y)	3.8 ± 1.5	3.7 ± 1.6	4.1 ± 1.2	0.780^*∗*^
8	Duration of injury (m)	4.1 ± 0.6	4.1 ± 0.5	4.3 ± 0.4	0.542^*∗*^

^*∗*^Nonsignificant.

**Table 2:** Comparison of pain intensity, sprint performance, and submaximal shuttle running of IKT, VRT, and control groups.

Sr. no.	Variable	IKT-G	VRT-G	Control-G	*p* value
1	Pain intensity				
	Baseline	7.3 ± 0.5	7.1 ± 0.6	7.3 ± 0.6	0.514^*∗*^
	4 weeks	4.8 ± 0.4	3.9 ± 0.5	6.2 ± 0.4	0.001^*∗∗*^
	8 weeks	2.7 ± 0.3	1.2 ± 0.4	5.4 ± 0.4	0.001^*∗∗*^
	6 months	1.9 ± 0.3	0.8 ± 0.4	4.2 ± 0.5	0.001^*∗∗*^
	*p* value	0.001^*∗∗*^	0.001^*∗∗*^	0.001^*∗∗*^	

2	Player wellness				
	Baseline	8.34 ± 1.3	8.52 ± 1.4	8.49 ± 1.5	0.944^*∗*^
	4 weeks	12.12 ± 1.2	14.43 ± 1.4	9.40 ± 1.4	0.001^*∗∗*^
	8 weeks	16.62 ± 1.4	18.05 ± 1.3	10.35 ± 1.3	0.001^*∗∗*^
	6 months	18.23 ± 1.4	20.79 ± 1.3	12.38 ± 1.5	0.001^*∗∗*^
	*p* value	0.001^*∗∗*^	0.001^*∗∗*^	0.001^*∗∗*^	

3	40 m sprint				
	Baseline	15.35 ± 0.2	15.48 ± 0.3	15.12 ± 0.3	0.541^*∗*^
	4 weeks	10.35 ± 0.2	8.12 ± 0.2	13.28 ± 0.3	0.001^*∗∗*^
	8 weeks	6.41 ± 0.3	5.25 ± 0.2	9.19 ± 0.1	0.001^*∗∗*^
	6 months	5.46 ± 0.1	4.81 ± 0.2	8.15 ± 0.3	0.001^*∗∗*^
	*p* value	0.001^*∗∗*^	0.001^*∗∗*^	0.001^*∗∗*^	

3	4 × 5 m sprint (s)				
	Baseline	23.81 ± 1.8	23.92 ± 1.6	23.19 ± 1.5	0.507^*∗*^
	4 weeks	18.26 ± 0.5	15.12 ± 0.5	19.09 ± 0.5	0.001^*∗∗*^
	8 weeks	12.22 ± 0.4	10.95 ± 0.5	17.08 ± 0.6	0.001^*∗∗*^
	6 months	8.96 ± 0.4	7.54 ± 0.3	15.01 ± 0.5	0.001^*∗∗*^
	*p* value	0.001^*∗∗*^	0.001^*∗∗*^	0.001^*∗∗*^	

4	Submaximal shuttle running A/P				
	Baseline	8.76 ± 2.2	7.93 ± 2.3	8.62 ± 2.4	0.643^*∗*^
	4 weeks	18.69 ± 2.5	20.33 ± 2.3	12.81 ± 2.5	0.001^*∗∗*^
	8 weeks	24.86 ± 2.8	28.02 ± 2.5	15.55 ± 2.1	0.001^*∗∗*^
	6 months	30.69 ± 2.2	32.67 ± 3.3	18.82 ± 3.4	0.001^*∗∗*^

	M/L				
	*p* value	0.001^*∗∗*^	0.001^*∗∗*^	0.001^*∗∗*^	
	Baseline	10.46 ± 3.2	11.53 ± 3.3	10.62 ± 3.2	0.685^*∗*^
	4 weeks	16.69 ± 3.2	19.33 ± 3.3	12.81 ± 3.5	0.001^*∗∗*^
	8 weeks	24.86 ± 3.3	26.02 ± 3.5	15.55 ± 3.2	0.001^*∗∗*^
	6 months	28.79 ± 3.2	32.67 ± 4.3	16.82 ± 3.9	0.001^*∗∗*^
	*p* value	0.001^*∗∗*^	0.001^*∗∗*^	0.001^*∗∗*^	

	Vertical				
	Baseline	16.62 ± 4.2	17.53 ± 3.8	17.62 ± 3.2	0.772^*∗*^
	4 weeks	24.69 ± 4.2	28.33 ± 4.3	20.81 ± 3.5	0.001^*∗∗*^
	8 weeks	36.86 ± 3.8	40.02 ± 3.5	24.55 ± 3.2	0.001^*∗∗*^
	6 months	45.45 ± 4.2	59.67 ± 4.3	30.82 ± 4.9	0.001^*∗∗*^
	*p* value	0.001^*∗∗*^	0.001^*∗∗*^	0.001^*∗∗*^	

^*∗*^Nonsignificant; ^*∗∗*^significant.

**Table 3:** Comparison of countermovement jump, squat jump, and player wellness of IKT, VRT, and control group.

Sr. no.	Variable	IKT-G	VRT-G	Control-G	*p* value
1	C jump height (cm)				
	Baseline	20.32 ± 2.4	21.52 ± 2.3	21.49 ± 2.5	0.388^*∗*^
	4 weeks	28.12 ± 2.5	32.45 ± 2.5	23.40 ± 2.4	0.001^*∗∗*^
	8 weeks	35.67 ± 3.4	40.05 ± 3.4	26.35 ± 3.5	0.001^*∗∗*^
	6 months	40.39 ± 3.3	46.79 ± 3.3	28.28 ± 3.4	0.001^*∗∗*^
	*p* value	0.001^*∗∗*^	0.001^*∗∗*^	0.001^*∗∗*^	
	Force (N)				
	Baseline	920.58 ± 120	910.4 ± 115	915.4 ± 112	0.977^*∗*^
	4 weeks	1013.1 ± 128	1112.4 ± 132	930.4 ± 118	0.001^*∗∗*^
	8 weeks	1182.6 ± 138	1286.5 ± 142	1072.3 ± 132	0.001^*∗∗*^
	6 months	1282.5 ± 185	1358.7 ± 180	1120.8 ± 172	0.001^*∗∗*^
	*p* value	0.001^*∗∗*^	0.001^*∗∗*^	0.001^*∗∗*^	
	Velocity (m·s^−1^)				
	Baseline	0.91 ± 0.02	0.91 ± 0.01	0.92 ± 0.02	0.277^*∗*^
	4 weeks	1.12 ± 0.03	1.45 ± 0.04	1.10 ± 0.04	0.001^*∗∗*^
	8 weeks	1.89 ± 0.06	2.05 ± 0.05	1.25 ± 0.03	0.002^*∗∗*^
	6 months	2.27 ± 0.02	2.79 ± 0.03	1.28 ± 0.04	0.001^*∗∗*^
	*p* value	0.001^*∗∗*^	0.001^*∗∗*^	0.001^*∗∗*^	

2	S jump height (cm)				
	Baseline	17.37 ± 2.4	17.52 ± 2.5	16.49 ± 2.3	0.531^*∗*^
	4 weeks	23.12 ± 1.5	28.45 ± 1.5	21.40 ± 1.4	0.193^*∗*^
	8 weeks	35.67 ± 1.4	38.05 ± 1.6	24.25 ± 1.5	0.002^*∗∗*^
	6 months	40.32 ± 2.4	43.79 ± 1.8	26.18 ± 1.9	0.001^*∗∗*^
	*p* value	0.001^*∗∗*^	0.001^*∗∗*^	0.001^*∗∗*^	
	Force (N)				
	Baseline	925.46 ± 95	932.52 ± 86	930.29 ± 92	0.981^*∗*^
	4 weeks	1087.12 ± 121	1187.25 ± 118	996.42 ± 123	0.193^*∗*^
	8 weeks	1258.57 ± 128	1327.55 ± 136	1098.3 ± 118	0.002^*∗∗*^
	6 months	1321.21 ± 135	1422.29 ± 152	1189.2 ± 112	0.001^*∗∗*^
	*p* value	0.001^*∗∗*^	0.001^*∗∗*^	0.001^*∗∗*^	
	Velocity (m·s^−1^)				
	Baseline	0.68 ± 0.02	0.69 ± 0.02	0.69 ± 0.03	0.891^*∗*^
	4 weeks	1.12 ± 0.04	1.45 ± 0.03	0.97 ± 0.02	0.193^*∗*^
	8 weeks	1.67 ± 0.03	2.15 ± 0.02	1.08 ± 0.03	0.002^*∗∗*^
	6 months	2.01 ± 0.04	2.47 ± 0.03	1.28 ± 0.4	0.001^*∗∗*^
	*p* value	0.001^*∗∗*^	0.001^*∗∗*^	0.001^*∗∗*^	

^*∗*^Nonsignificant; ^*∗∗*^significant.

## Data Availability

The master data used to support the findings of this study have not been made available because of medicolegal issues of the country.

## References

[1] Micheli L. J., Wood R. (1995). Back pain in young athletes. *Archives of Pediatrics & Adolescent Medicine*.

[2] Vos T., Allen C., Arora M. (2016). Global, regional, and national incidence, prevalence, and years lived with disability for 310 diseases and injuries, 1990–2015: a systematic analysis for the global burden of disease study 2015. *Lancet*.

[3] Ruhe A., Fejer R., Walker B. (2011). Is there a relationship between pain intensity and postural sway in patients with non-specific low back pain?. *BMC MusculoskeletDisord*.

[4] Silsupadol P., Siu K.-C., Shumway-Cook A., Woollacott M. H. (2006). Training of balance under single- and dual-task conditions in older adults with balance impairment. *Physical Therapy*.

[5] Ferreira M. L., Sherrington C., Smith K. (2012). Physical activity improves strength, balance and endurance in adults aged 40–65 years: a systematic review. *Journal of Physiotherapy*.

[6] Frobell R. B., Roos H. P., Roos E. M., Roemer F. W., Ranstam J., Lohmander L. S. (2013). Treatment for acute anterior cruciate ligament tear: five year outcome of randomised trial. *BMJ*.

[7] Emery C. A., Meeuwisse W. H. (2010). The effectiveness of a neuromuscular prevention strategy to reduce injuries in youth soccer: a cluster-randomised controlled trial. *British Journal of Sports Medicine*.

[8] Nambi G., Kamal W., Shanmugananath E. S. (2018). Spinal manipulation plus laser therapy versus laser therapy alone in the treatment of chronic non-specific low back pain: a randomized controlled study. *European Journal of Physical and Rehabilitation Medicine*.

[9] Thorborg K., Krommes K. K., Esteve E., Clausen M. B., Bartels E. M., Rathleff M. S. (2017). Effect of specific exercise-based football injury prevention programmes on the overall injury rate in football: a systematic review and meta-analysis of the FIFA 11 and 11+ programmes. *British Journal of Sports Medicine*.

[10] Finch C. F., Doyle T. L., Dempsey A. R. (2014). What do community football players think about different exercise-training programmes? Implications for the delivery of lower limb injury prevention programmes. *British Journal of Sports Medicine*.

[11] Ho C.-W., Chen L.-C., Hsu H.-H. (2005). Isokinetic muscle strength of the trunk and bilateral knees in young subjects with lumbar disc herniation. *Spine*.

[12] Dvir Z., Keating J. (2003). Trunk extension effort in patients with chronic low back dysfunction. *Spine*.

[13] Storheim K., Holm I., Gunderson R., Brox J. I., Bø K. (2003). The effect of comprehensive group training on cross-sectional area, density, and strength of paraspinal muscles in patients sick-listed for subacute low back pain. *Journal of Spinal Disorders & Techniques*.

[14] Nambi G., Abdelbasset W. K., Alqahtani B. A., Alrawaili S. M., Abodonya A., Saleh A. K. (2020). Isokinetic back training is more effective than core stabilization training on pain intensity and sports performances in football players with chronic low back pain: a randomized controlled trial. *Medicine*.

[15] Gil-Gómez J.-A., Lloréns R., Alcañiz M., Colomer C. (2011). Effectiveness of a Wii balance board-based system (eBaViR) for balance rehabilitation: a pilot randomized clinical trial in patients with acquired brain injury. *Journal of NeuroEngineering and Rehabilitation*.

[16] Lange B. S., Requejo P., Flynn S. M. (2010). The potential of virtual reality and gaming to assist successful aging with disability. *Physical Medicine and Rehabilitation Clinics of North America*.

[17] Mao Y., Chen P., Li L., Huang D. (2014). Virtual reality training improves balance function. *Neural Regeneration Research*.

[18] Yesilyaprak S. S., Yildirim M. S., Tomruk M. (2016). Comparison of the effects of virtual reality-based balance exercises and conventional exercises on balance and fall risk in older adults living in nursing homes in Turkey. *Physiother Theory Practice*.

[19] Yang W.-C., Wang H.-K., Wu R.-M., Lo C.-S., Lin K.-H. (2016). Home-based virtual reality balance training and conventional balance training in Parkinson’s disease: a randomized controlled trial. *Journal of the Formosan Medical Association*.

[20] Shih M. C., Wang R. Y., Cheng S. J. (2016). Effects of a balance-based exergaming intervention using the Kinect sensor on posture stability in individuals with Parkinson’s disease: a single-blinded randomized controlled trial. *Journal of NeuroEngineering and Rehabilitation*.

[21] Filiz S., Sibel E., Hale K. (2009). Comparison of isokinetic exercise versus standard exercise training in patients with chronic low back pain: a randomized controlled study. *Clinical Rehabilitation*.

[22] Wi S.-Y., Kang J.-H. (2012). The effects of virtual reality interactive games on the balance ability of elderly women with knee osteoarthritis. *Journal of the Korean Society of Physical Medicine*.

[23] Shahbandar L., Press J. (2005). Diagnosis and nonoperative management of lumbar disk herniation. *Operative Techniques in Sports Medicine*.

[24] Ferraz M. B., Quaresma M. R., Aquino L. R. (1990). Reliability of pain scales in the assessment of literature and illiterate patients with rheumatoid arthritis. *The Journal of Rheumatology*.

[25] Hooper S. L., Mackinnon L. T. (1995). Monitoring overtraining in athletes. *Sports Medicine*.

[26] Sporis G., Jukic I., Milanovic L., Vucetic V. (2010). Reliability and factorial validity of agility tests for soccer players. *Journal of Strength and Conditioning Research*.

[27] Boyd L. J., Ball K., Aughey R. J. (2011). The reliability of MinimaxX accelerometers for measuring physical activity in Australian football. *International Journal of Sports Physiology and Performance*.

[28] Glatthorn J. F., Gouge S., Nussbaumer S., Stauffacher S., Impellizzeri F. M., Maffiuletti N. A. (2011). Validity and reliability of optojump photoelectric cells for estimating vertical jump height. *Journal of Strength and Conditioning Research*.

[29] Calmes P., Jacob J. F., Fayolle-Minon I. (2004). Use of isokinetic techniques vs Standard physiotherapy in patients with chronic low back pain. Preliminary results. *Annales de Réadaptation et de Médecine Physique*.

[30] Ben Moussa Zouita A., Ben Salah F. Z., Dziri C., Beardsley C. (2018). Comparison of isokinetic trunk flexion and extension torques and powers between athletes and nonathletes. *Journal of Exercise Rehabilitation*.

[31] Zouita A. B. M., Zouita S., Dziri C., Brughelli M., Behm D. G., Chaouachi A. (2019). Differences in trunk strength between weightlifters and wrestlers. *Journal of Human Kinetics*.

[32] Hibbs A. E., Thompson K. G., French D., Wrigley A., Spears I. (2008). Optimizing performance by improving core stability and core strength. *Sports Medicine*.

[33] Van Damme B. B. L., Stevens V. K., Van Tiggelen D. E., Duvigneaud N. N. P., Neyens E., Danneels L. A. (2013). Velocity of isokinetic trunk exercises influences back muscle recruitment patterns in healthy subjects. *Journal of Electromyography and Kinesiology*.

[34] Morini S., Ciccarelli A., Cerulli C., Giombini A., Di Cesare A., Ripani M. (2008). Functional anatomy of trunk flexion-extension in isokinetic exercise: muscle activity in standing and seated positions. *The Journal of Sports Medicine and Physical Fitness*.

[35] Almuzaini K. S., Potteiger J. A., Green S. B. (1998). Effects of split exercise sessions on excess postexercise oxygen consumption and resting metabolic rate. *Canadian Journal of Applied Physiology*.

[36] Gleeson M., Bishop N. C., Stensel D. J., Lindley M. R., Mastana S. S., Nimmo M. A. (2011). The anti-inflammatory effects of exercise: mechanisms and implications for the prevention and treatment of disease. *Nature Reviews Immunology*.

[37] McConville K. M. V., Virk S. (2012). Evaluation of an electronic video game for improvement of balance. *Virtual Reality*.

[38] D’hooge R., Cagnie B., Crombez G. (2012). Increased intramuscular fatty infiltration without differences in lumbar muscle cross sectional area during remission of unilateral recurrent low back pain. *Manual Therapy*.

[39] Danneels L. A., Vanderstraeten G. G., Cambier D. C., Witvrouw E. E., De Cuyper H. J., Danneels L. (2000). CT imaging of trunk muscles in chronic low back pain patients and healthy control subjects. *European Spine Journal*.

